# Genome size analyses of Pucciniales reveal the largest fungal genomes

**DOI:** 10.3389/fpls.2014.00422

**Published:** 2014-08-26

**Authors:** Sílvia Tavares, Ana Paula Ramos, Ana Sofia Pires, Helena G. Azinheira, Patrícia Caldeirinha, Tobias Link, Rita Abranches, Maria do Céu Silva, Ralf T. Voegele, João Loureiro, Pedro Talhinhas

**Affiliations:** ^1^Centro de Investigação das Ferrugens do Cafeeiro, BioTrop, Instituto de Investigação Científica TropicalOeiras, Portugal; ^2^Plant Cell Biology Laboratory, Instituto de Tecnologia Química e Biológica António Xavier, Universidade Nova de LisboaOeiras, Portugal; ^3^CEER-Biosystems Engeneering, Instituto Superior de Agronomia, Universidade de LisboaLisbon, Portugal; ^4^Department of Life Sciences, Centre for Functional Ecology, University of CoimbraCoimbra, Portugal; ^5^Institut für Phytomedizin, Universität HohenheimStuttgart, Germany

**Keywords:** flow cytometry, *Gymnosporangium confusum*, mycological cytogenomics, nuclear DNA content, rust fungi

## Abstract

Rust fungi (Basidiomycota, Pucciniales) are biotrophic plant pathogens which exhibit diverse complexities in their life cycles and host ranges. The completion of genome sequencing of a few rust fungi has revealed the occurrence of large genomes. Sequencing efforts for other rust fungi have been hampered by uncertainty concerning their genome sizes. Flow cytometry was recently applied to estimate the genome size of a few rust fungi, and confirmed the occurrence of large genomes in this order (averaging 225.3 Mbp, while the average for Basidiomycota was 49.9 Mbp and was 37.7 Mbp for all fungi). In this work, we have used an innovative and simple approach to simultaneously isolate nuclei from the rust and its host plant in order to estimate the genome size of 30 rust species by flow cytometry. Genome sizes varied over 10-fold, from 70 to 893 Mbp, with an average genome size value of 380.2 Mbp. Compared to the genome sizes of over 1800 fungi, *Gymnosporangium confusum* possesses the largest fungal genome ever reported (893.2 Mbp). Moreover, even the smallest rust genome determined in this study is larger than the vast majority of fungal genomes (94%). The average genome size of the Pucciniales is now of 305.5 Mbp, while the average Basidiomycota genome size has shifted to 70.4 Mbp and the average for all fungi reached 44.2 Mbp. Despite the fact that no correlation could be drawn between the genome sizes, the phylogenomics or the life cycle of rust fungi, it is interesting to note that rusts with Fabaceae hosts present genomes clearly larger than those with Poaceae hosts. Although this study comprises only a small fraction of the more than 7000 rust species described, it seems already evident that the Pucciniales represent a group where genome size expansion could be a common characteristic. This is in sharp contrast to sister taxa, placing this order in a relevant position in fungal genomics research.

## Introduction

**Figure 1:**
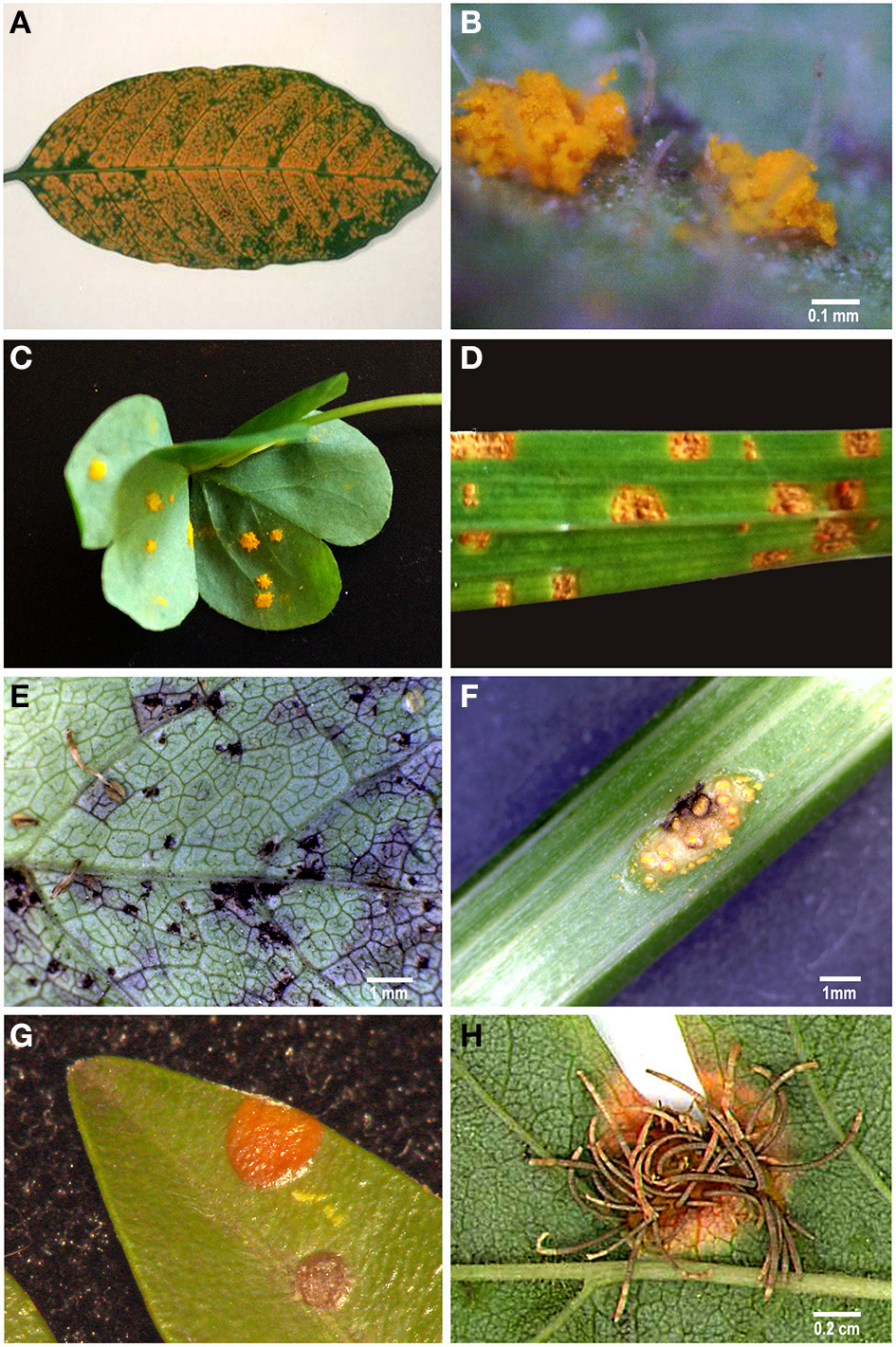
**Examples of rust sporulation. (A)** *Hemileia vastatrix* uredinia on a *Coffea arabica* leaf; **(B)** *Phragmidium mexicanum* uredinia on a *Duchesnea indica* leaf; **(C)** *Puccinia oxalidis* uredinia on a *Oxalis articulata* leaf; **(D)** *Uromyces transversalis* uredinia on a *Gladiolus* sp. leaf; **(E)** *Puccinia smyrnii* telia on a *Smyrnium olusatrum* leaf; **(F)** *Puccinia smyrnii* aecia on a *Smyrnium olusatrum* stem; **(G)** *Puccinia buxi* telia on a *Buxus sempervirens* leaf; **(H)** *Gymnosporangium confusum* aecia on a *Crataegus monogyna* leaf.

The Pucciniales (Fungi, Basidiomycota, Pucciniomycotina) represent the largest group of fungal plant pathogens. They are characterized by orange, brown or red colored spore masses (Figure 1) appearing on the host tissue surface. Rust fungi are obligate biotrophs, depending entirely on living host cells to complete their biological cycle (Cummins and Hiratsuka, 2003). Their life cycles are diverse, both in terms of the number of spore types produced (micro-, hemi-, demi-, or macrocyclic) and their requirement (or not) of alternate hosts for life cycle completion (autoecious or heteroecious) (for a recent review see Fernandez et al., 2013). Karyogamy occurs in teliospores that germinate to produce basidia, the structure where meiosis takes place. Teliospores are thus responsible for sexual reproduction (Aime, 2006). Rust fungi are generally highly specialized pathogens frequently having narrow host ranges, and consequently they share a common evolutionary history with their host plants (Duplessis et al., 2011b). Rust fungi are able to infect plants from most families, including conifers, ferns and mosses, and are responsible for major diseases on agricultural and forest crops worldwide. Rust epidemics have impacted the development of human society, such as the early accounts of cereal rusts coming from the Bible and from Greek and Roman literatures (Park and Wellings, 2012), or the reports of coffee leaf rust epidemics in Sri Lanka in the 19th century (Silva et al., 2006).

Genome sequencing of some rust species provided evidence for their large genome sizes (Cantu et al., 2011; Duplessis et al., 2011a) especially when compared to non-biotrophic fungi (Spanu, 2012). Genome sequencing in additional rust species confirms this (Nemri et al., 2014; Tan et al., 2014). Nevertheless, sequencing efforts of other rusts species have been hampered by uncertainty concerning the genome size of the species of sequencing interest. Genome size records for 11 rust species (mostly from *Puccinia, Melampsora* and *Uromyces* genera) can be found at the Fungal Genome Size database (Kullman et al., 2005) and in the literature (Supplementary Data). With an average of 225.3 Mbp, available genome size values of rust species range from 77 Mbp (*Cronartium quercuum* f. sp. *fusiforme* Burds., and G.A. Snow; Anderson et al., 2010) to 733 Mbp (*Hemileia vastatrix* (733 Mbp; Carvalho et al., 2014).

Although considerably smaller than most other eukaryotes, fungi exhibit a remarkable variation in their genome sizes. The average fungal genome size is 37.7 Mbp overall, and 49.9 Mbp for the Basidiomycota (Kullman et al., 2005). The two largest fungal genomes reported so far are those of *Neottiella vivida* (Nyl.) Dennis (Ascomycota, Pezizales; Kullman, 2002) and *Scutellospora castanea* Walker (Glomeromycota, Diversisporales; Zeze et al., 1996; Hijri and Sanders, 2005), with 750 and 795 Mbp/1C, respectively. Variations in chromosome number and size are far from being an exception and ploidy levels ranging from 1x to 50x have already been found (Gregory et al., 2007). Nevertheless, Basidiomycota cells are more frequently dikaryotic with haploid nuclei for most of their life cycles. Such variations are often considered to be adaptive (Kelkar and Ochman, 2012), since variations in genome size of plant pathogens can have a direct impact in their pathogenicity (D'Hondt et al., 2011). This occurs namely through the diversity-creating effect of the activity of transposable elements and/or of polyploidization, or through the presence (or absence) of supernumerary/dispensable chromosomes (Aguileta et al., 2009; Albertin and Marullo, 2012).

Most probably due to technical constraints related with their smaller genome sizes in comparison with other organisms, only in the last two decades flow cytometry was considered the method of choice for genome size determination studies in fungi, with important impacts on plant pathology (D'Hondt et al., 2011). Using this technique, the size of the genome is estimated by comparing the fluorescence emitted by an intercalating DNA fluorochrome of a sample together with a reference standard with known genome size. Given that a flow cytometer is available, the method provides reliable estimates of genome size in a very short period of time (10 min.) and can be considered a fast and relatively cheap alternative to other molecular tools (D'Hondt et al., 2011). Still, rust species may pose technical constraints in the determination of genome sizes, as it can be especially difficult to extract nuclei in good quantity and quality from spores of obligate parasites.

Therefore, the objective of this study was to elucidate the apparent genome size expansion in the Pucciniales suggested by the genome size information available for a few species, by addressing a larger number of rust fungi with distinct life cycles, hosts and types of spores produced. For such, this work describes an innovative approach for obtaining nuclear suspensions from fungi, in particular from obligate parasites, such as the rusts. The chopping procedure of Galbraith et al. (1983) developed for plant tissues was applied for the first time for plant pathogenic fungi, enabling the analyses to be carried out directly on infected samples and not on spores.

## Materials and methods

### Biological material

A total of 23 rust samples were obtained from field surveys (during 2013 and 2014 in the Lisbon area, Portugal) as infected plant material, being subsequently identified by microscopic observation. Infected plant material was preserved as dry herbarium specimens at the “João de Carvalho e Vasconcellos” Herbarium (LISI; Lisbon, Portugal). Another nine samples were retrieved as urediniospores from active collections. Thus, 32 rust samples were subjected to analysis, as detailed in Table 1. Infected plant material was employed directly for fungal (and plant) nuclear isolation and subsequently for flow cytometric analysis. Urediniospores (ca. 50 mg) were spread on sterile water in Petri dishes and incubated over-night at 25°C to obtain germ tubes, or used directly for flow cytometry.

**Table 1:**
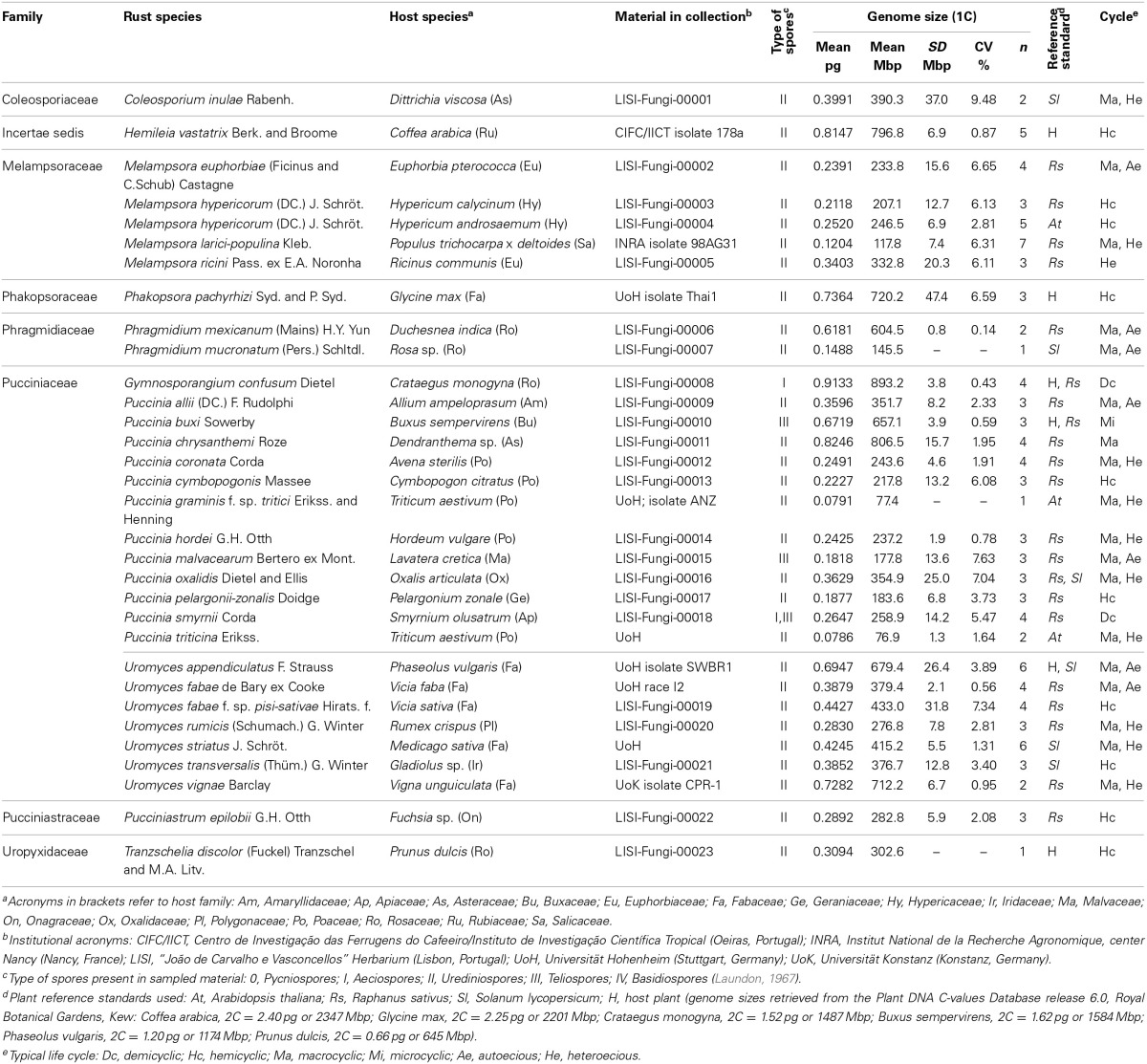
**List of 32 rust samples analyzed for genome size determination, with reference to (and sorted by) family and species, source of material [host plant (botanical name, family, location and infection stage), or spores in collection], plant reference standard used, average (GS, in pg, and Mbp), standard deviation (SD, in Mbp) and coefficient of variation (CV, in %) of the monoploid genome size, number of samples (n) and typical life cycle**.

*^a^Acronyms in brackets refer to host family: Am, Amaryllidaceae; Ap, Apiaceae; As, Asteraceae; Bu, Buxaceae; Eu, Euphorbiaceae; Fa, Fabaceae; Ge, Geraniaceae; Hy, Hypericaceae; Ir, Iridaceae; Ma, Malvaceae; On, Onagraceae; Ox, Oxalidaceae; Pl, Polygonaceae; Po, Poaceae; Ro, Rosaceae; Ru, Rubiaceae; Sa, Salicaceae*.

*^b^Institutional acronyms: CIFC/IICT, Centro de Investigação das Ferrugens do Cafeeiro/Instituto de Investigação Científica Tropical (Oeiras, Portugal); INRA, Institut National de la Recherche Agronomique, center Nancy (Nancy, France); LISI, “João de Carvalho e Vasconcellos” Herbarium (Lisbon, Portugal); UoH, Universität Hohenheim (Stuttgart, Germany); UoK, Universität Konstanz (Konstanz, Germany)*.

*^c^Type of spores present in sampled material: 0, Pycniospores; I, Aeciospores; II, Urediniospores; III, Teliospores; IV, Basidiospores (Laundon, 1967)*.

*^d^Plant reference standards used: At, Arabidopsis thaliana; Rs, Raphanus sativus; Sl, Solanum lycopersicum; H, host plant (genome sizes retrieved from the Plant DNA C-values Database release 6.0, Royal Botanical Gardens, Kew: Coffea arabica, 2C = 2.40 pg or 2347 Mbp; Glycine max, 2C = 2.25 pg or 2201 Mbp; Crataegus monogyna, 2C = 1.52 pg or 1487 Mbp; Buxus sempervirens, 2C = 1.62 pg or 1584 Mbp; Phaseolus vulgaris, 2C = 1.20 pg or 1174 Mbp; Prunus dulcis, 2C = 0.66 pg or 645 Mbp)*.

*^e^Typical life cycle: Dc, demicyclic; Hc, hemicyclic; Ma, macrocyclic; Mi, microcyclic; Ae, autoecious; He, heteroecious*.

Plants used as reference for flow cytometry were grown from seeds (*Arabidopsis thaliana* ‘Col-0,’ *Raphanus sativus* ‘Saxa’ and *Solanum lycopersicum* ‘Stupické’) and were maintained at the CFE/FCT/UC.

### Fluorescence microscopy

Fungal material was stained using an aqueous solution of 1 μg/ml 4′,6-diamidino-2-phenylindole (DAPI; Sigma-Aldrich, St. Louis, USA) and slides were mounted in vectashield® (Vector Laboratories, Burlingame, USA), an antifading agent. Samples were observed in an epifluorescence microscope (Leica DMRB, DFC 340FX) equipped with a BP470/40 cube and an excitation wavelength of 340–380 nm. Pictures were captured with MetaMorph® software.

Infected leaf pieces, about 2–4 cm^2^, were fixed overnight in a 2% solution of glutaraldehyde in 0.1 M sodium phosphate buffer, pH 7.2. Leaf pieces were then sectioned with a freezing microtome (Leica CM1850) and the sections (20–25 μm) were stained with DAPI (as before), for 2 h. The sections were then washed with distilled water, stained with an aqueous solution of 0.3% w/v diethanol for 2–3 s, washed again with distilled water and mounted in 50% v/v glycerol (adapted from Stark-Urnau and Mendgen, 1993; Skalamera and Heath, 1998).

Leaf material was examined with bright field microscopes (Leitz Dialux 20 and Leica DM-2500) equipped with mercury bulbs HB 100W, ultra-violet light (excitation filter BP 340–380; barrier filter LP 430).

### Flow cytometry

The nuclear DNA content of rust fungi was estimated by flow cytometry using infected host tissue samples (occasionally spores or germ tubes only), by comparison with the host plant genome size (as given in Table 1) and/or, when the latter was either unknown, uncertain or out of range, with healthy leaves of plant DNA reference standards: *Arabidopsis thaliana* ‘Col-0’ (2C = 0.32 pg or 313 Mbp; this study after calibration with *Raphanus sativus* ‘Saxa’); *Raphanus sativus* ‘Saxa’ (2C = 1.11 pg or 1086 Mbp; Doležel et al., 1992); or *Solanum lycopersicum* ‘Stupické’ (2C = 1.96 pg or 1917 Mbp; Doležel et al., 1992) (Table 1).

Nuclei were released from infected host tissues, fungal germ tubes and/or leaves of the reference standards following the procedure of Galbraith et al. (1983). In brief, ~50 mg of both fungus and plant (internal standard) were chopped with a razor blade in a Petri dish with 1 mL of Woody Plant Buffer (WPB; 0.2 M Tris–HCl, 4 mM MgCl_2_, 1% Triton X-100, 2 mM Na_2_EDTA, 86 mM NaCl, 20 mM sodium metabisulfite, 1% PVP-10, pH 7.5; Loureiro et al., 2007). Nuclei from spores were released by grinding ~10 mg of spores in a mortar in the presence of 1 mL of WPB. For the latter, nuclei from the plant DNA reference standard were added afterwards (pseudo-internal standardization).

The nuclear suspension was then filtered through a 30 μm nylon filter to remove plant and fungal debris, and 50 μg/mL of propidium iodide (PI; Fluka, Buchs, Switzerland) and 50 μg/mL of RNase (Fluka), both suspended in water, were added to stain the DNA only. After incubation for 5 min. at room temperature, the fluorescence intensity of at least 3000 nuclei per sample was analyzed using a Partec CyFlow Space flow cytometer (Partec GmbH, Görlitz, Germany), equipped with a 30 mW green solid-state laser emitting at 532 nm for optimal PI excitation. The assignment of each peak to rust fungi, host plant and plant reference standard was confirmed by separately analysing healthy plant samples and fungal spores or germ tubes. For each rust species, the G_1_ peak of the plant species used as internal reference standard was set to a specific channel (usually between channel positions 500 and 750 on a 0-1028 scale), with the amplification system kept at a constant voltage and gain throughout the analyses. Each day, prior to analysis, the overall instrument quality was assessed using calibration beads green concentrate (Partec GmbH). For each sample, when possible at least three independent replicate measurements were performed.

### Flow cytometry data analysis

Data were acquired using Partec FloMax software v2.4d (Partec GmbH) in the form of four graphics: fluorescence pulse integral in linear scale (FL); forward light scatter (FSC) vs. side light scatter (SSC), both in logarithmic (log) scale; time vs. FL in linear scale; and SSC in log scale vs. FL in linear scale. To analyse only intact nuclei, the FL histogram was gated with a polygonal region defined in the FL vs. SSC dot-plot (Loureiro et al., 2006). Afterwards, using FloMax gating tools, linear regions were created in the FL histogram to gate the nuclei and obtain descriptive statistics of each peak, including number of nuclei, mean channel position and coefficient of variation (CV).

The genome size in mass units (1C in pg for fungi) was assessed using the formula:

$$\frac{\begin{array}{c} \text{Mean G1 fluorescence of}\\ \text{sample nuclei} \end{array}}{\begin{array}{c} \text{Mean G1 fluorescence of}\\ \text{reference standard} \end{array}}\times \begin{array}{c} \text{2C genome size of the}\\ \text{reference standard} \end{array}$$

Conversion of mass values into numbers of base pairs was done according to the factor 1 pg = 978 Mbp (Doležel and Bartoš, 2005).

The reliability of the genome size measurements was verified by evaluating the quality of the flow cytometry histograms based on the CV of the G_1_ peaks and on the background debris, and by the CV of the genome size estimation of each isolate based on the independent measurements. According with the criteria established by Bourne et al. (2014), only CV values of DNA peaks below 10% were considered in the analyses.

### Statistical analysis

Statistical analyses were performed using R (R Core Team, 2014). Comparison of genome size values for the most outstanding phylogenetic groups was performed using the Wilcoxon test (α = 0.05). Comparison of individual data was performed using the χ^2^-test. A total of 1820 fungal genome sizes were compiled from information publically available at the Fungal Genome Size Database (http://www.zbi.ee/fungal-genomesize; Kullman et al., 2005), the JGI Genome Portal (http://genome.jgi-psf.org), the Broad Institute (http://www.broadinstitute.org/), and from the literature (Supplementary Data, database sheet).

## Results

**Figure 2:**
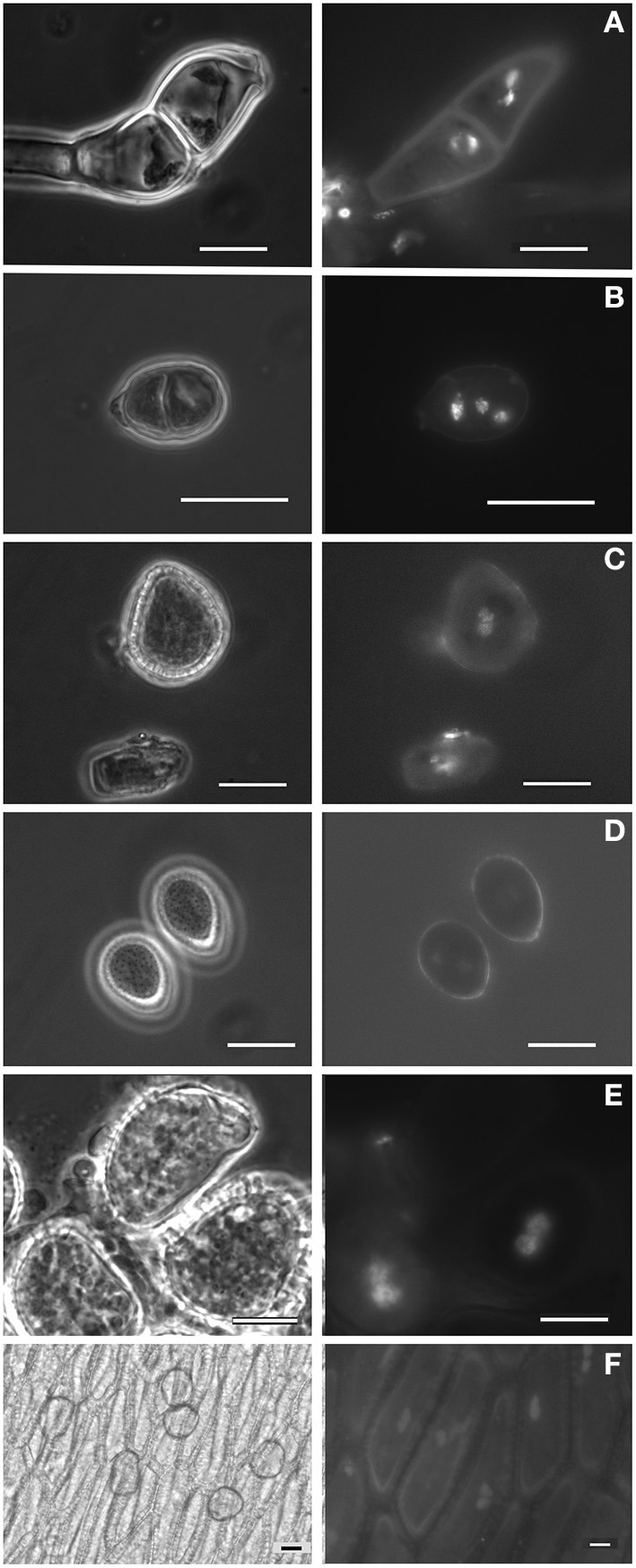
**Spores (and other cells) from Pucciniales under phase-contrast microscopy and showing the DAPI-stained nuclei under fluorescence light. (A)** Teliospore from *Puccinia malvacearum*; **(B)** Teliospore from *Puccinia smyrnii*; **(C)** Aeciospore from *Puccinia smyrnii*; **(D)** Urediniospores from *Phragmidium mexicanum*; **(E)** Urediniospore from *Hemileia vastatrix*; **(F)** Pseudoperidia cells and aeciospores from *Gymnosporangium confusum*. Bars, 10 μm.

**Figure 3:**
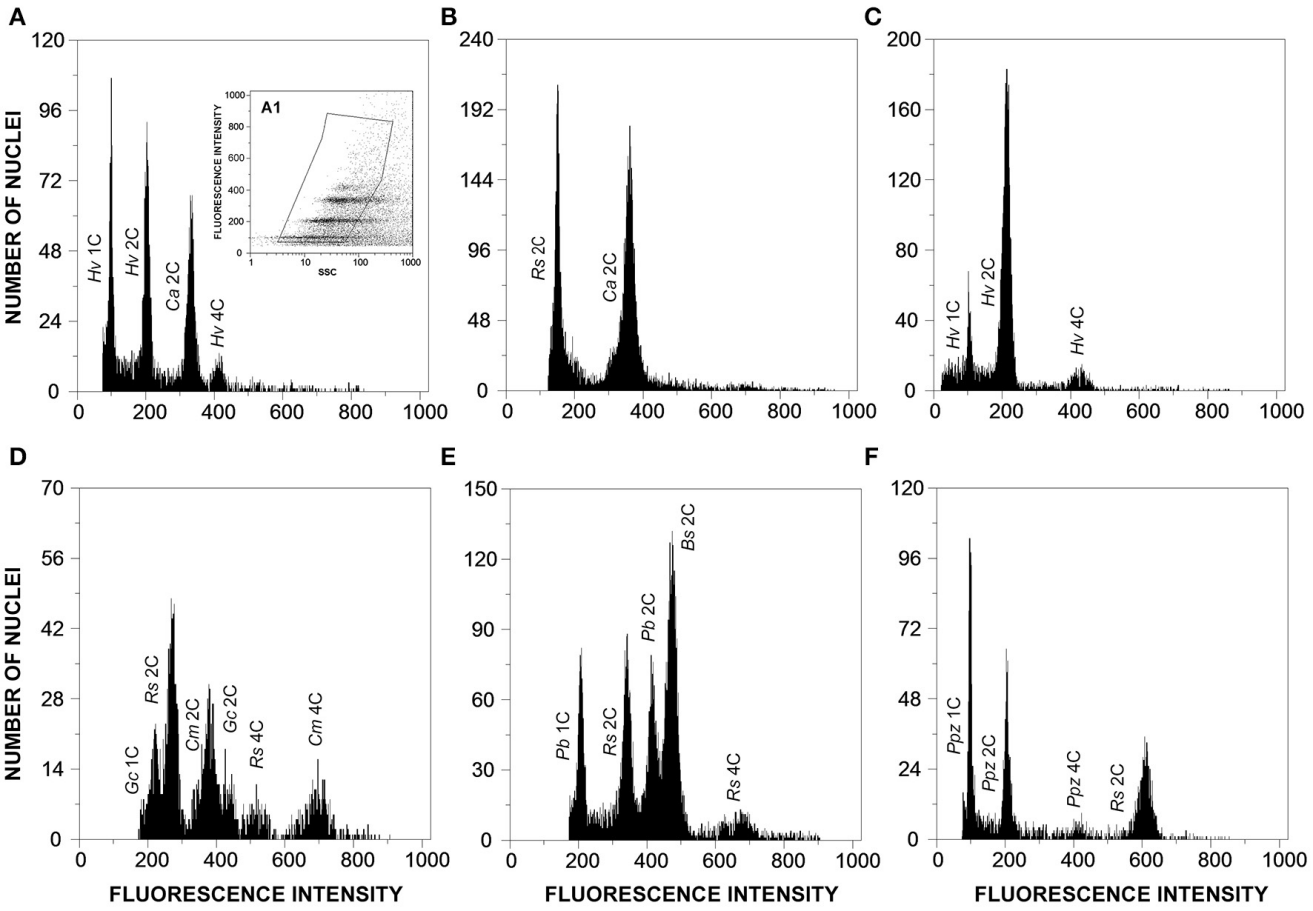
**Flow cytometric histograms of relative fluorescence intensities of propidium iodide-stained nuclei simultaneously isolated from: (A)** *Hemileia vastatrix* (*Hv*) and its host plant, *Coffea arabica* (*Ca*; 2C = 2.49 pg DNA); **(B)** *Coffea arabica* (Ca) and the plant DNA reference standard, *Raphanus sativus* (*Rs*, 2C = 1.11 pg DNA); **(C)** *Hemileia vastatrix* (*Hv*) hyphae obtained upon germination of urediniospores in water; **(D)** *Gymnosporangium confusum* (*Gc*), its host plant, *Crataegus monogyna* (*Cm*; 2C = 1.50 pg DNA), and the plant DNA reference standard, *Raphanus sativus* (*Rs*); **(E)** *Puccinia buxi* (*Pb*), the plant DNA reference standard (*Rs*), and its host plant, *Buxus sempervirens* (*Bs*; 2C = 1.60 pg DNA); and **(F)** *Puccinia pelargonii-zonalis* (*Ppz*) and the plant DNA reference standard, *Raphanus sativus* (*Rs*). The inset (A1) in histogram A represents the gating made in the dot-plot of SSC vs. FL to exclude as much as possible partial nuclei and other types of debris.

Field surveys conducted over 1 year enabled the identification of several rust-infected plants. Both plants and fungi were identified by experienced botanists and mycologists. The collected samples (Table 1) comprised several botanical and mycological families. Most rusts were retrieved at the urediniosporic infection cycle, but telia and aecia were also readily found for certain rusts (Figures 1, 2), further confirming the diagnosis and allowing the identification of the pathogen. Staining of spores with DAPI enabled the visualization of two nuclei per rust cell in aeciospores and urediniospores. In teliospores, some cells contained two nuclei, while others, following karyogamy, contained a single nucleus (Figure 2). The application of the nuclear isolation protocol to rust-infected host tissues (Figure 1) enabled the release of intact nuclei from both the host plant and the fungal cells. Nuclei were efficiently stained with PI, according to the clearly defined G_1_ peaks of both organisms (Figure 3). Following this innovative approach, after identification of the fungal peaks, 32 rust samples representing 30 species were analyzed by flow cytometry (Table 1).

The genome size determinations based on the fungal G_1_ fluorescence peaks had CV values below 10% (usually between 4 and 7%), which is within the range of accepted values for fungal species (Bourne et al., 2014), and, for each sample, CV measures of genome size estimations never exceeded 10% (Table 1). Polygonal regions in dot-plots of SSC vs. FL enabled to gate and present in a histogram nuclei that were uniform in size and shape, eliminating partial nuclei and other types of debris (Figure 3A1). This strategy improved the CV values of DNA peaks and high-quality histograms were obtained for the analyses of genome sizes.

When the genome size of the host plant was known and appropriate (i.e., when it appeared in the same scale set as the fungal species), the host plant itself was used as primary reference standard. Otherwise, according with the genome size of the fungal species, *Arabidopsis thaliana, Raphanus sativus*, or *Solanum lycopersicum* were used as reference genomes. The analysis was not affected by the endopolyploid nature of *R. sativus* and especially of *A. thaliana* (Kudo and Kimura, 2001) since the only visible peak of plant DNA reference standard in the scale set was that of 2C nuclei and thus the three plant species were considered adequate for the analysis. Even when the rust and the host genome sizes were within the same size range, the host genome was always larger than that of the rust (as exemplified in Figures 3A,D,E).

In this study we have analyzed 12 *Puccinia* spp., six *Uromyces* spp., four *Melampsora* spp., and two *Phragmidium* spp. The remaining six genera analyzed were represented by a single species (Table 1). The average genome size of species of *Melampsora, Puccinia* and *Uromyces* was 227.6, 303.6, and 467.5 Mbp respectively. While the five *Melampsora* genomes (four species) were all below the overall average and varied by less than 3x, from 117.8 Mbp (for *M. larici-populina*) to 332.8 Mbp (for *M. ricini*), the 11 *Puccinia* species varied by more than 10x, and included the smallest genomes analyzed in this study (76.9 and 77.4 Mbp for *P. triticina* and *P. graminis* f. sp. *tritici*) and the second largest one, *P. chrysanthemi* with 806.5 Mbp. The seven *Uromyces* genomes (six species) varied also by less than 3x, but in most cases their genome sizes were higher than the overall average, from 276.8 Mbp in *U. rumicis* to 712.2 Mbp in *U. vignae*. No statistically significant differences were identified when comparing rust genera or families.

When rusts are clustered according to their hosts family, it is clear that rusts with Poaceae hosts (*Puccinia coronata, P. cymbopogonis, P. graminis* f. sp. *tritici, P. hordei, and P. triticina*) have considerably smaller genomes (170.6 Mbp on average) than rusts with Fabaceae hosts (556.6 Mbp on average; *Phakopsora pachyrhizi, Uromyces appendiculatus, U. fabae, U. fabae* f. sp. *pisi-sativae, U. striatus*, and *U. vignae*). This difference is statistically supported (*P* < 0.05). The four rust species with Rosaceae hosts (*Gymnosporangium confusum, Phragmidium mexicanum, Phr. mucronatum*, and *Tranzschelia discolor*) presented vastly different genome sizes, with estimates below the average (145.5 Mbp in *Phr. mucronatum*) to the largest estimate discovered so far (893.2 Mbp in *G. confusum*), with an average of 486.4 Mbp.

**Figure 4:**
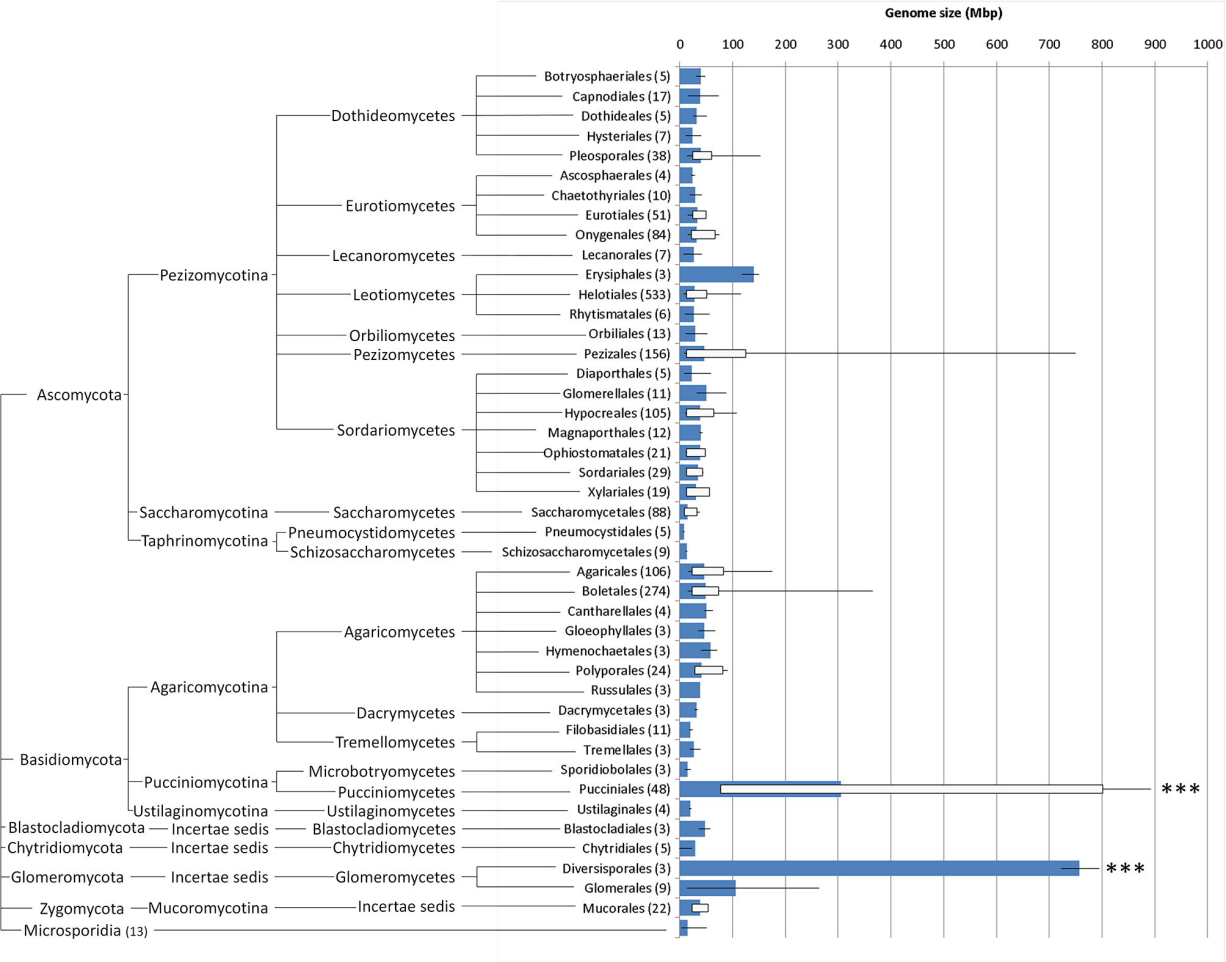
**Whisker box plots for genome sizes (Mbp) for every fungal order for which at least three values were available (number of organisms in brackets; further details in Supplementary Data, database sheet), including the results obtained in this study; blue bars represent average, lines denote minimum and maximum values and white boxes represent 5 and 95 percentiles; fungal orders are arranged phylogenetically (http://tolweb.org/Fungi/)**. ^***^P < 0.001.

Adding these values to the available genome sizes for other Pucciniales (Supplementary Data, database sheet) results in a global average for all rust fungi of 305.5 Mbp, a value that is significantly higher (*P* < 0.001) than that of any other order in Fungi for which three or more genome sizes are available (Figure 4), with the single exception of the Diversisporales (Glomeromycota), which contains three genomes in the 723–795 Mbp range. The smallest rust genome size in this list (*Cronartium quercuum* f. sp. *fusiforme*, with 76.6 Mbp; Anderson et al., 2010) is larger than 94% of all the fungi analyzed so far. The global average for all fungi (including the results obtained in this work) is of 44.2 Mbp (genome sizes from 1852 organisms), a value that is to a large extent influenced by the large amount of estimations available for the Ascomycota (1278 organisms, i.e., 69% of all organisms; global average of 31.8 Mbp), and Basidiomycota fungi (516 organisms; 28%; global average of 70.4 Mbp (Supplementary Data, analysis sheet).

## Discussion

The Pucciniales represent an important group of plant pathogens, with several unifying biological characteristics. Recently, the occurrence of a genome size expansion in the Pucciniales was suggested based on the first completed rust genome sequences (Spanu, 2012). With the purpose of providing a broader set of data to support or refute this hypothesis, the genome size of 32 rust samples (comprising 30 rust species) was estimated by flow cytometry. The chopping procedure of fresh tissue for isolating intact nuclei in the presence of a nuclear isolation buffer (developed for plant tissues) was successfully employed for flow cytometric analysis of the genome sizes of rust-infected plant material. This approach proved to be very efficient and may be applied on a variety of infected plant tissues in the future, as it circumvents the need to isolate basidiospores or pycniospores as previously reported for flow cytometric estimation of genome size in rust fungi (Williams and Mendgen, 1975; Eilam et al., 1992, 1994).

A collection of rust fungi found in nature, together with some of the economically most important rust species, revealed that the variability of the genomes sizes was very high, ranging from 76.9 to 893.2 Mbp. These estimates are even higher than those already made available through the fungal genome size database. Two rust fungi, *Puccinia chrysanthemi* and *Gymnosporangium confusum*, with genome sizes of 806.5 and 893.2 Mbp/1C respectively, constitute the two largest fungal genomes reported to date. Both genomes are larger than the so far largest rust genome, *Hemileia vastatrix* (Carvalho et al., 2014 and in the present study). These genome sizes also surpass the two largest fungal genomes reported so far, *Neottiella vivida* and *Scutellospora castanea*. Remarkably both of these fungi also interact closely with plants.

Comparing the results obtained in this study with the 1820 fungal genome sizes available in databases and in the literature, it is evident that even the smallest rust genome is larger than the genome size found in 94% of all fungi. The inclusion of these 32 genome sizes shifts the global genome size average of all fungi from 37.7 to 44.2 Mbp, and that of Basidiomycota from 49.9 to 70.4 Mbp. The average genome size for the Pucciniales reaches 305.5 Mbp, a value that is significantly higher than any other Ascomycota or Basidiomycota order. The few genome sizes available for species of other orders in the Pucciniomycotina besides the Pucciniales are much smaller, with estimates of 13 Mbp for *Mixia osmundae* (Nishida) C.L. Kramer, of 26 Mbp for *Microbotryum violaceum* (Pers.) G. Deml and Oberw. and of 21 Mbp for *Rhodotorula graminis* Di Menna and *Sporobolomyces roseus* Kluyver and C.B. Niel.

The collection of rusts under study represents different hosts and life cycles and comprises 10 rust genera, enabling the analysis of correlations between these characteristics and the genome size estimates obtained. The two *Phragmidium* species analyzed in this study differ clearly in their genomes sizes, despite both being macrocyclic and autoecious. *Phragmidium mexicanum* infects *Potentilla/Duchesnea* hosts (Yun et al., 2011), while *Phr. mucronatum* colonizes species of *Rosa* (Helfer, 2005). Two *Melampsora hypericorum* samples obtained from *Hypericum calycinum* or *H. androseamum* also exhibited distinct genome sizes. The latter supports other reports that have shown the occurrence of host-depended intra-specific variation in nuclear content of *Puccinia hordei* and of *P. recondita* isolates (Eilam et al., 1994).

The genome size of *Puccinia graminis* f. sp. *tritici* is estimated to be 77.4 Mbp. Eilam et al. (1994) estimated a value of 67 Mbp, while the genome sequence yielded a value of 88.6 Mbp (Duplessis et al., 2011a). These differences could also be attributed to intra-specific variability, although the distinct methodologies adopted may also account for some variation. A difference of 16.7 Mbp was observed between the flow cytometric estimate and the value obtained from genome sequencing (Duplessis et al., 2011a) for *Melampsora larici-populina* isolate 98AG31. *Uromyces appendiculatus* and *U. vignae* have been reported to have some of the largest rust genomes (Eilam et al., 1994; Kullman et al., 2005), with 400 to 418 Mbp. In this work, however the genome size of laboratory strains of these two species was estimated as 679.4 and 712.2 Mbp, respectively. Such discrepancies could be due to the employment of a different technique.

All *Uromyces* species analyzed with Fabaceae hosts presented genome sizes above 300 Mbp. Moreover, *Phakopsora pachyrhizi*, another rust that infects a member of the Fabaceae, also possesses a large genome size. This markedly contrasts with the smaller genome sizes of rusts with Poaeceae hosts (all below 250 Mbp and all in the genus *Puccinia*). Interestingly, *Uromyces* species with Fabaceae hosts constitute a monophyletic group that probably evolved together, and are all autoecious (van der Merwe et al., 2008). Considering the relationship between genome sizes and life cycles, the species with the largest genome sizes are either autoecious or hemicyclic with no known alternate host, with the exception of the heteroecious *Gymnosporangium confusum*.

As a microcyclic rust, *Puccinia buxi* depends strictly in sexual reproduction for multiplication. This fungus is only found in a limited number of locations, most likely due to its specific requirements of shaded and humid microclimatic conditions (Preece, 2000; Durrieu, 2001), thus suggesting low population size. In this study, this species was also found to possess a large genome, which could be linked to its populational and reproductive characteristics. In fact, a major force conditioning genome size seems to be genetic drift, which was negatively correlated with effective population size (Kelkar and Ochman, 2012).

The Pucciniales share some common features, such as biotrophy and obligate parasitism. Biotrophy has been highlighted as a lifestyle that leads to increasing genome size as compared to non-biotrophs (Spanu, 2012). The very large genome sizes of the 30 rust fungal species revealed by our study strongly reinforce the view that expanded genome sizes occur among biotrophs, and that large genomes are a common characteristic of the Pucciniales. From the genome sequencing of rust fungi (e.g., Duplessis et al., 2011a; Nemri et al., 2014) and other biotrophs it is now clear that larger genomes do not imply higher numbers of structural genes, resulting invariably in an increased proliferation of transposable elements (TE) and repetitive DNA. Such a genomic environment can create genetic polymorphisms, especially in the case of sexual abstinence (Spanu, 2012). As in plants, it would be interesting to evaluate in the future if there are costs for the fungi associated with the accumulation and replication of this excess DNA (large genome constraint; for a review see Knight et al., 2005).

Although the effect of sex on genome size evolution is still unclear (Raffaele and Kamoun, 2012), three of the rust fungi with large genome sizes, *Hemileia vastatrix, Phakospora pachyrhizi* and to some extent *Puccinia chrysanthemi*, all rely on asexual reproduction. The first two species are hemicyclic or at least the aecial host is unknown and the third also reproduces mainly asexually, although it was reported to be autoecious in Japan (Alaei et al., 2009). Even for those species which are capable of sexual reproduction, it is expectable that urediniosporic infection cycles may well represent a very important fraction of reproduction, for which TE activity would be potentially an important source for the generation of diversity. In this sense, rust species that do not produce urediniospores (demicyclic rusts), such as the autoecious *Puccinia buxi* and *P. smyrnii*, and therefore strictly depend on sexual reproduction for life cycle completion, are of great interest for studying the relation between genome size and reproduction/diversity creation strategies.

A very large fraction (up to 50%) of the rust genome sequences published so far is composed of repetitive elements. Those genomes, however, are all below 200 Mbp. In this work we have revealed genome sizes several times larger. One can speculate that such genome size expansions could be due to an even higher proportion of non-coding regions, but also to genome duplication/polyploidy. Although still largely overlooked in fungi, polyploidy is a major evolutionary process in eukaryotes (Albertin and Marullo, 2012), playing a role on the wide capacity of fungi to evolve adaptability to virtually all ecosystems and modes of heterotrophic nutrition (Aguileta et al., 2009). Polyploidy events may have occurred in other Basidiomycota (such as the Agaricales), and tolerance for genome merging has been suggested in the Microbotryales (Pucciniomycotina) (Albertin and Marullo, 2012). Although genome sizes in the Pucciniales are clearly expanded as compared to neighboring clades, variation in genome sizes across the Pucciniales suggests little correlation to phylogeny. *Hemileia vastatrix*, which was considered to represent an ancestral clade in rusts phylogeny (Aime, 2006), presents one of the largest genome sizes determined in this study. Also, species within the same genus (e.g., in *Puccinia* or *Uromyces*) presented very divergent genome sizes. These finds suggest that variation in genome sizes is rapidly occurring along the evolution of Pucciniales.

*Gymnosporagium* spp. are unique rust fungi since they comprise the only genus forming teliospores on members of the Cupressaceae. Molecular data (18S and 28S rDNA sequences) question their placement within the Pucciniaceae (Aime, 2006). Now due to its highest genome size, this group is likely to gain more attention from the scientific community.

A unifying characteristic amongst the species with a larger genome size within the Pucciniales was not found. It seems more likely that different events have driven the evolution of genome size of particular species or groups of species. Genome variability is considered to be adaptive and host driven resulting in a high capability to overcome the host defenses (Stukenbrock and Croll, 2014). Relationships between genome size and biological parameters are of special interest because they can be linked to the ability of an organism to overcome selection pressure (D'Hondt et al., 2011). Although the rust genome sizes determined in this study surpass most other fungi and are within the range of genome sizes of many plants, it is interesting to note that all rust genome sizes in this study are smaller than those of the hosts from where they were obtained.

In conclusion, in this work the analysis of the genome size of 30 rust species (representing eight families) revealed the occurrence of very large genome sizes, including the two largest fungal genomes ever reported, *Gymnosporangium confusum* (893.2 Mbp) and *Puccinia chrysanthemi* (806.5 Mbp). Although comprising only a very small fraction of the more than 7000 rust species described, with many genera and some families not represented, this work suggests that the Pucciniales represent a group where genome size expansion could be a common characteristic, in sharp contrast with sister taxa, making this group of organisms a subject of utmost interest for genomic research and for further studies.

## Author contributions

This study was conceived and directed by Sílvia Tavares, Ana Paula Ramos, Ana Sofia Pires, Helena G. Azinheira, Tobias Link, Rita Abranches, Ralf T. Voegele, João Loureiro, and Pedro Talhinhas. Collection and identification of field material was performed by Ana Paula Ramos, Helena G. Azinheira, and Pedro Talhinhas. Sample preparation, nuclei isolation and flow cytometry analyses were performed by Sílvia Tavares, Patrícia Caldeirinha, João Loureiro, and Pedro Talhinhas. Microscopy observations and image acquisition were conducted by Sílvia Tavares, Ana Sofia Pires, and Maria do Céu Silva. Data analysis and biological interpretation of results were conducted by Sílvia Tavares, Ana Paula Ramos, João Loureiro, and Pedro Talhinhas. Sílvia Tavares, Ana Paula Ramos, João Loureiro, and Pedro Talhinhas wrote the paper. All authors read and approved the final manuscript.

### Conflict of interest statement

The authors declare that the research was conducted in the absence of any commercial or financial relationships that could be construed as a potential conflict of interest.
